# Prescription Function Prediction Using Topic Model and Multilabel Classifiers

**DOI:** 10.1155/2017/8279109

**Published:** 2017-10-11

**Authors:** Lidong Wang, Yin Zhang, Yun Zhang, Xiaodong Xu, Shihua Cao

**Affiliations:** ^1^Qianjiang College, Hangzhou Normal University, Hangzhou 310018, China; ^2^College of Computer Science and Technology, Zhejiang University, Hangzhou, Zhejiang 310027, China; ^3^Zhejiang University of Media and Communications, Hangzhou, Zhejiang 310018, China; ^4^Zhejiang Chinese Medical University, Hangzhou, Zhejiang 310053, China

## Abstract

Determining a prescription's function is one of the challenging problems in Traditional Chinese Medicine (TCM). In past decades, TCM has been widely researched through various methods in computer science, but none concentrates on the prediction method for a new prescription's function. In this study, two methods are presented concerning this issue. The first method is based on a novel supervised topic model named Label-Prescription-Herb (LPH), which incorporates herb-herb compatibility rules into learning process. The second method is based on multilabel classifiers built by TFIDF features and herbal attribute features. Experiments undertaken reveal that both methods perform well, but the multilabel classifiers slightly outperform LPH-based method. The prediction results can provide valuable information for new prescription discovery before clinical test.

## 1. Introduction

Traditional Chinese Medicine (TCM) is a unique medical knowledge system in China and has become a popular complementary treatment in Western countries. Currently there are 100,000 formulae based on the continuous clinical records. A formula is a prescription that is validated by pharmacology and clinics. Researchers have made great efforts to study and utilize those formulae to discover new prescriptions hidden in the formulae data [1]. To discover a new prescription for disease treatment, researchers have to analyze the efficiency of related herbs and collect several herbs with proper proportion according to TCM theory. Then, the function of a new prescription has to be proved through repeated clinical tests, which would require a large amount of manpower and material resources. Actually, if a new prescription's function can be prepredicted by computer science technology, the results would provide valuable reference for the following clinical practices.

It has been found that data mining approaches play critical roles in TCM related topics, such as new drug discovery [1], syndrome differentiation [2–4], herbal combinational rule mining [5, 6], symptom name normalization [7], intelligent diagnosis [8], and treatment pattern mining [9]. Most of the previous research was related to relationship mining, such as herb-symptom relationships [8, 10, 11] and herb-herb relationships [6]. Wang et al. [6] created a herbal network to present the herb-herb correlation. Chen et al. [8] detected the patterns between herbs and symptoms by using tripartite information network. Recently, more and more researchers have adopted topic models to mine the correlation between TCM objects. Lin et al. [10] proposed a symptom-herb-therapies-diagnosis topic model to diagnose the disease and administer appropriate drugs and treatments given a patient's symptoms. Zhang et al. [4] proposed a Symptom-Herb-Diagnosis Topic (SHDT) model to extract multiple relationships among symptoms, herb combinations, and diagnoses from large-scale CM clinical data. The proposed model was useful in discovering the common TCM diagnosis and treatment patterns. Jiang et al. [11] applied Linked LDA to extract the herb-symptom patterns. Yao et al. [9] employed Labeled LDA (Labeled Latent Dirichlet Allocation) to mine treatment patterns in TCM clinical cases, but the mining result was not satisfactory. Unlike these studies, we concentrate on the prescription function prediction through topic detection and incorporate compatibility rule mining into the topic model.

In TCM theory, a prescription's function can be affected mainly by the following factors: the attributes of herbs, the compatibility rules of paired herbs, and the dosages. Based on this, we present two methods to predict a prescription's function. The first method is based on topic modeling. A novel topic model named LPH (Label-Prescription-Herb) is proposed to incorporate the results of compatibility rule mining into learning process. It can automatically learn the posterior distribution of each herb in a prescription conditioned on the prescription's label set (function set). The second method is based on feature extraction and multilabel classifiers. We extract *N*-dimensional feature vector space for each prescription concerning their herbal attributes and TFIDF (Term Frequency-Inverse Document Frequency) Features and then employ several popular and competitive classifiers to validate our method.

The rest of paper is organized as follows.  Section 2 presents the detailed steps of our methods for prescription function prediction.  Section 3 provides analyses and discussion of our experimental results. Finally, some conclusions and future works are provided in Section 4.

## 2. Methods

**Figure 1:**
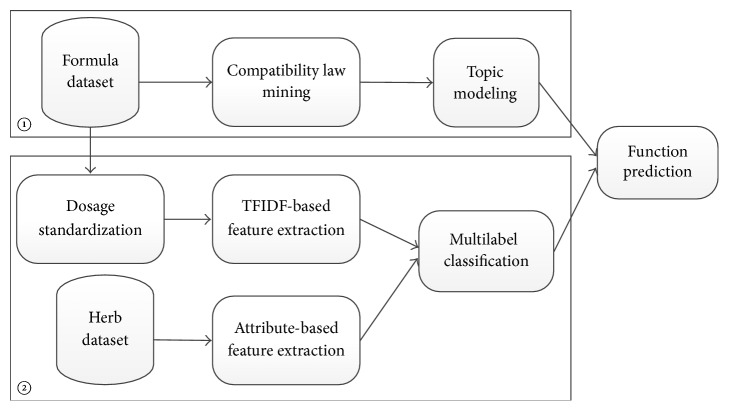
The framework of our methods.

The framework of our methods is shown in Figure 1, with details presented in following subsections.

The herb dataset and formula dataset are extracted from our project CKCEST (http://zcy.ckcest.cn/tcm/) (Chinese Knowledge Center for Engineering Science and Technology). In the first method, we conduct compatibility rule mining from the formula dataset and then incorporate the results into the learning process of topic modeling. The objective of topic modeling is to learn the “topic-word” (function-herb) structure with supervision. The prescription's most likely labels can then be inferred by thresholding its posterior probability over function labels. In the second method, we treat our prediction task as a multiclass, multilabel classification problem. We extract feature space based on TFIDF weighting and herbal attributes and then train the multilabel classification model by using the features.

### 2.1. Prediction Based on Topic Model

In this section, we propose a supervised topic model named Label-Prescription-Herb (LPH) to mine treatment patterns in the herbs of the formula dataset. Although a prescription consists of two or more individual herbs, some of them act as pairs in the treatment. In this subsection we introduce the method to mine the compatibility rules.

#### 2.1.1. Compatibility Rule Mining

In TCM theory, compatibility refers to the combination of two or more herbs based on the clinical settings and the properties of herbs [12]. The efficiency of a single herb is usually limited, but when two herbs are used together, their interaction should display their superiority over a single herb in the treatment of diseases; we say that these two herbs have compatibility rule. In China, many herbs have intensive compatibility rule that have been learned from ancient times to the modern period. However, the existing 917 herb pairs in Chinese Paired Herb Database are inadequate for our prediction task. Thus, computer intelligence can be employed to discover more pairs for further research. When two herbs are frequently used in combination with each other, they are more likely to be paired drugs. We propose a method based on support degree [13] and dependency relationship for compatibility rule mining between herb *h*_*i*_ and herb *h*_*j*_, which is consists of the following steps:

**Step 1 .**

(1) $$\begin{array}{c} support=p\left(\;h_{i},h_{j}\;\right). \end{array}$$

**Step 2 .**

(2) $$\begin{array}{c} dependency=\frac{p\left(h_{i},h_{j}\right)}{p\left(h_{i}\right)p\left(h_{j}\right)}. \end{array}$$

**Step 3 .**

(3) $$\begin{array}{c} Cor=a\cdot support+b\cdot dependency. \end{array}$$

**Step 4 .**

Rank all possible herb pairs according to their associated value of Cor.

**Step 5 .**

Return top-*N* pairs.

Here support denotes the joint probability of occurrence of two herbs *h*_*i*_ and *h*_*j*_. In Step 3, we combine the support attribute (*p*(*h*_1_, *h*_2_)) and the dependency attribute (the ratio of *p*(*h*_1_, *h*_2_) to *p*(*h*_1_)*p*(*h*_2_)). Note that we remove* Glycyrrhizae Radix* from the mining results, since it is useless to analyze compatibility rule between* Glycyrrhizae Radix* and other herbs. The use of this herb is merely in decreasing or moderating medicinal side-effects of all herbs in a prescription.

#### 2.1.2. Topic Model Description on TCM

LDA (Latent Dirichlet Allocation) is a completely unsupervised method that models each document as a mixture of topics [14]. The model outputs a discrete probability distribution over words for each topic and a discrete distribution over topics for each document. However, LDA is not appropriate for multilabeled corpora because it generates automatic summaries of topics that have no direct correspondence with the label set. A simple solution to this problem is to assign a document's words to its labels rather than to a latent and possibly less interpretable semantic space. At present there exists some related research, such as Labeled LDA [15] and partially Labeled LDA [16].

Analogous to the relationship among documents, topics, and words, we can treat herbs as “words.” A prescription (formula) is a bag of herbs, and we can treat it as a structured “document.” Correspondingly, a prescription's function can be considered as a “topic.” Thus, we employ topic models to mine the latent relationship between function labels and herbs. The topic model for our prediction task should incorporate supervision by constraining the model to use only those “topics” that correspond to a prescription's label set. Since the combination of herbs contributes a factor to the function prediction, we consider the role of herb pairs in the topic learning process.

We define some notations. Let each prescription *p* be represented by a tuple consisting of a list of herbs, **H** ^(*p*)^ = {*h*_1_, *h*_2_,…, *h*_*N*_*p*__} and a list of binary topic presence/absence indicators Λ^(*p*)^ = {*l*_1_, *l*_2_,…, *l*_*K*_}, where each *h*_*i*_ ∈ {1,…, *V*} and each *l*_*k*_ ∈ {0,1}. Here *N*_*p*_ is the prescription length, *V* is the total number of herbs extracted from formula dataset and *K* is the total number of function labels. We set the number of functions in our model to be the number of unique labels *K*.

#### 2.1.3. LPH Model

**Figure 2:**
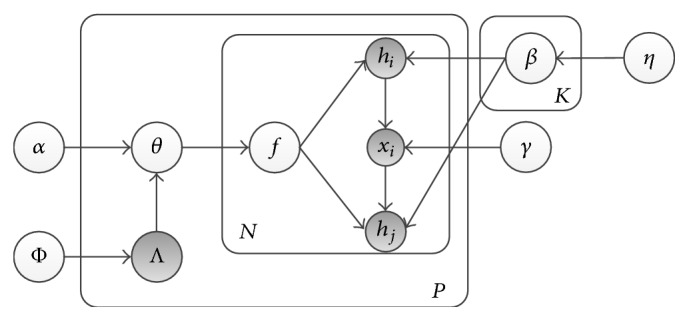
Graphical model of improved Labeled LDA.

To incorporate compatibility rules into the topic model, we introduce variable *x*_*i*_ to indicate whether herb *h*_*i*_ has compatibility rule with herb *h*_*j*_. If *x*_*i*_ = 1, then *h*_*i*_ and *h*_*j*_ are paired herbs; otherwise, they are generated from the distribution associated with their function label. The graphical model of LPH model is shown in Figure 2.

In Figure 2, *β*_*k*_ is a vector consisting of the parameters of multinomial distribution corresponding to the *k*th function label. *γ*_*i*_ is the prior parameter for variable *x*_*i*_. *α* are the parameters of the Dirichlet topic prior and *η* are the parameters of the herb prior, while Φ_*k*_ is the label prior for function *k*. The generative process for LPH model is given as follows:

1. For each function *k* ∈ [1,…, *K*], generate *β*_*k*_ from a Dirichlet distribution with prior parameter *η*, that is, *β*_*k*_ ~ Dir(*η*).
2. For each prescription *p*:
  - For each function *k* ∈ [1,…, *K*], generate function label (topic) presence/absence indicators Λ_*k*_ from a Bernoulli distribution with prior parameter Φ_*k*_, that is, Λ_*k*_ ~ Bernoulli(Φ_*k*_).
  - Generate the parameters of the Dirichlet function prior ${\vec{\alpha }}^{\left(p\right)}$ from the label projection matrix **L** and the predefined Dirichlet priors $\vec{\alpha }$, that is, ${\vec{\alpha }}^{\left(p\right)}=L\times \vec{\alpha }$.
  - Generate function mixture *θ* from Dirichlet distribution $Dir({\vec{\alpha }}^{\left(p\right)})$, that is, $\theta \sim Dir({\vec{\alpha }}^{\left(p\right)})$.
3. For each herb *h*_*i*_, *i* ∈ {1,…, *N*_*p*_}:
  - Generate *x*_*i*_ from Bernoulli distribution Bernoulli (*γ*_*i*_), that is, *x*_*i*_ ~ Bernoulli(*γ*_*i*_).
  - Generate function *f* from multinomial distribution Mult(*θ*), that is, *f* ~ Mult(*θ*).
  - If *x*_*i*_ = 0, generate a herb *h*_*i*_ from multinomial distribution Mult(*β*_*f*_), that is, *h*_*i*_ ~ Mult(*β*_*f*_); if *x*_*i*_ = 1, generate herb pair (*h*_*i*_, *h*_*j*_) from multinomial distribution Mult(*β*_*f*_), that is, (*h*_*i*_, *h*_*j*_) ~ Mult(*β*_*f*_).

During step (2)(b), label projection matrix **L** is used to project the Dirichlet prior vector $\vec{\alpha }=\{\alpha_{1},\ldots ,\alpha_{K}\}$ into a lower dimension ${\vec{\alpha }}^{\left(p\right)}$. For instance, suppose *K* = 6 and that a prescription *p* has labels given by Λ^(*p*)^ = (0,0, 0,1, 1,0) which implies **L** would be

(4) $$\begin{array}{c} \left(\begin{array}{cccccc} 0 & 0 & 0 & 1 & 0 & 0\\ 0 & 0 & 0 & 0 & 1 & 0 \end{array}\right). \end{array}$$

The *i*th row of **L** has an entry of 1 in column *j* if and only if the *i*th label in prescription *p* is equal to the function *j* and 0 otherwise. Then, function mixture *θ* is drawn from a Dirichlet distribution with parameters ${\vec{\alpha }}^{\left(p\right)}=L\times \vec{\alpha }=(\alpha_{4},\alpha_{5}{)}^{T}$.

During step (3)(a), when the parameter *x*_*i*_ for the herb *h*_*i*_ is observed from the compatibility rule mining results, the prior parameter *γ*_*i*_ is separated from the rest of the models. Analogous to Labeled LDA, for prescription *p*, we restrict *θ* to be defined over topics corresponding to its prior labels Λ^(*p*)^. This restriction ensures that all the topic assignments are limited to the prescription's labels.

#### 2.1.4. Learning and Inference

The exact inference for LPH is intractable, thus several approximate schemes have been proposed to infer the model. We use collapsed Gibbs sampling [17] to estimate the probability of a function label *k* assigned to the herb *h*_*i*_ in a prescription. We first choose initial states for the Markov chain randomly; then we calculate the conditional distribution *p*(*f*_*i*_ = *k*∣**f**_−*i*_) and *p*(*f*_(*i*, *j*)_ = *k*∣**f**_−*i*,−*j*_) as follows, where **f**_−*i*_ denotes all herbs' function label assignments excluding *h*_*i*_; **f**_−*i*,−*j*_ denotes all herbs' function label assignments excluding *h*_*i*_ and *h*_*j*_.

(5) If  xi=0,pfi=k ∣ f−i∝n−i,khi+ηhin−i,k·+ηT1→×n−i,kp+αkn−i,·p+αT1→

(6) If  xi=1,pfi,j=k ∣ f−i,−j∝n−i,khi+ηhin−j,khj+ηhjn−i,−j,k·+ηT1→×n−i,−j,kp+αkn−i,−j,·p+αT1→.

In (5), *n*_−*i*,*k*_^*h*_*i*_^ is the count of herb *h*_*i*_ in function *k*, *n*_−*i*,*k*_^(·)^ is the total number of herbs assigned to function *k*, *n*_−*i*,*k*_^*p*^ is the number of times herbs in prescription *p* are assigned to function *k*, and *n*_−*i*,·_^*p*^ is the number of herbs in *p*. All counts exclude the current assignment. In (6), all counts do not include the current two cases *h*_*i*_ and *h*_*j*_. Note that once a herb pair (*h*_*i*_, *h*_*j*_) is assigned to the function *k*, the two herbs *h*_*i*_ and *h*_*j*_ will be assigned to the topic simultaneously.

After Gibbs sampling iterations, we estimate the function-herb multinomial distribution *β* and the prescription function mixture *θ* as follows:

If *x*_*i*_ = 0, then

(7) θpk=n−i,kp+αkn−i,·p+αT1→,βkhi=n−i,khi+ηhin−i,k·+ηT1→.

If *x*_*i*_ = 1, then

(8) θpk=n−i,−j,kp+αkn−i,−j,·p+αT1→,βkhi,hj=n−i,khi+ηhin−j,khj+ηhjn−i,−j,k·+ηT1→.

#### 2.1.5. Function Prediction

During multilabel prediction, inferring the best set of labels for an unlabeled prescription at test time is more complex: it involves assessing all function label assignments and returning the assignment that has the highest posterior probability. However, the issue is not so simple, since there are 2^*K*^ possible function label assignments. For the purpose of this paper, we infer the conditional probability of function labels (topics) given a new prescription by using Bayes rules (see (9)). The prescription's most probable labels can then be inferred by suitably thresholding its posterior probability over function labels. Suppose a new prescription *p* consists of a set of herbs **H** ^(*p*)^ = {*h*_1_, *h*_2_,…, *h*_*N*_*p*__}, then *p*(*k*∣**H** ^(*p*)^) is calculated as follows:

(9) pk ∣ Hp∏hi,hj∈Hpphi ∣ kpkxi=0·phi,hj ∣ kpkxi=1=∏hi,hj∈Hpβkhipkxi=0·βkhi,hjpkxi=1.

To simplify calculation, *p*(*k*) can be treated as a constant and *p*(*k*∣**H** ^(*p*)^) can be calculated as follows:

(10) $$\begin{array}{c} p\left(\;k\;|\;H^{\left(p\right)}\;\right)\propto \underset{h_{i},h_{j}\in H^{\left(p\right)}}{\prod }\beta_{k}{\left(\;h_{i}\;\right)}_{\left\{x_{i}=0\right\}}\cdot \beta_{k}{\left(\;h_{i},h_{j}\;\right)}_{\left\{x_{i}=1\right\}}. \end{array}$$

### 2.2. Feature Extraction

In this section, we adopt the TFIDF method and herbal attributes to extract a prescription's features.

#### 2.2.1. TFIDF Features

TFIDF is often used as a weighting factor in information retrieval and text mining. In TCM, some herbs appear frequently to tend to have little influence on a prescription's function, such as* Glycyrrhizae Radix*. In this work, we employ TFIDF to reflect the importance of a herb for a prescription in a collection. A prescription is treated as a “document,” and the corresponding herbs are treated as “terms.” So, we denote TF(*h*_*i*_) = *F*(*h*_*i*_), which is the frequency of *h*_*i*_ and define IDF(*h*_*i*_) = log(*N*/*F*′(*h*_*i*_)), where *N* is the number of prescriptions; *F*′(*h*_*i*_) = |{*j* : *h*_*i*_ ∈ *p*_*j*_}| is the number of prescriptions containing the herb *h*_*i*_. Then, the TFIDF feature for the herb *h*_*i*_ can be denoted as follows:

(11) $$\begin{array}{c} TFIDF\left(\;h_{i}\;\right)=F\left(\;h_{i}\;\right)\log\left(\;\frac{N}{F^{\prime }\left(h_{i}\right)}\;\right). \end{array}$$

Based on this, we use the TFIDF features to represent a prescription:

(12) $$\begin{array}{c} \vec{p}=\left\{\;t_{1},t_{2},\ldots ,t_{m}\;\right\}, \end{array}$$

where *t*_*i*_ = TFIDF(*h*_*i*_) if the prescription contains herb *h*_*i*_, otherwise 0. *m* is the total number of unique herbs.

However, a prescription contains no information about the number of occurrences for each herb. Thus, we cannot calculate *F*(*h*_*i*_) this way. To solve this problem, we set the herb's dosage as its initial weight. The dosage information can reflect the importance of a herb in a prescription but should be standardized before our task, since different herbs have different usual dosages. For instance, the usual dosage for* Pseudoginseng* is 3 g ~ 9 g, while that of* Dioscoreae Rhizoma* is 15 g~ 30 g. So, the dosage of herbs in a prescription may not be directly comparable. For a prescription, we first standardize each herb's dosage before the TFIDF weighting phase by the following rule:

(13) $$\begin{array}{c} d_{i}^{*}=\frac{d_{i}}{d_{max}+d_{min}}, \end{array}$$

where *d*_*i*_ is the actual dosage of herb *h*_*i*_ in a prescription, *d*_max_ is its maximum usual dosage, and *d*_min_ is the minimum usual dosage.  Table 1 shows an example of dosage standardization on prescription “Ma Huang Tang.” The standardized dosage keeps the order of original data; that is, if a herb has higher dose in prescription *p*_*A*_ than in prescription *p*_*B*_, it remains in the same order after standardization. Then, *F*(*h*_*i*_) can be calculated as

(14) $$\begin{array}{c} F\left(\;h_{i}\;\right)=\frac{d_{i}^{*}}{\sum_{j=1}^{N_{p}}d_{j}^{*}}. \end{array}$$

#### 2.2.2. Attribute Features

The attributes of each herb, named “channel tropism,” “nature & flavor,” and “efficiency,” are described with certain terms. For instance, “nature” refers to the temperature characteristics of the herb, such as “cold,” “hot,” and “warm.” “Flavor” refers to the taste property of the herb, such as “sour,” “bitter,” and “sweet.”

For each prescription, we sort the herbs according to its *F*(*h*_*i*_) and select top two herbs to represent the prescription. For the herb *h*_*i*_, we collect 9 attributes in “nature & flavor,” 12 attributes in “channel tropism,” and 46 attributes in “efficiency.” Then, the attribute feature vector for a prescription can be denoted as $\vec{V}=\{v_{1},v_{2},\ldots ,v_{m}\}$, where *m* = 134, *v*_*i*_ ∈ [0,1]. If a herb contains feature *i*, the corresponding *v*_*i*_ is 1, otherwise 0. Some specific attributes, such as “slightly bitter” and “slightly hot,” are quantified as 0.5.

We consider our prediction task as a multilabel classification problem: given a training set consisting of prescriptions with multiple function labels, predict the set of labels appropriate for each prescription in the test set. Based on the above features, several multiple one-vs-rest classifiers are trained to test our method. These classifiers are SVM (Support Vector Machine), Adaboost, and Bayes Network, which are popular and extremely competitive baselines used by most previous papers [18].

## 3. Results

We collected 3055 formulae (https://github.com/violetconch/label-prescription-herb-model) and 972 herbs for our experiments, the former were derived from our project CKCEST (http://zcy.ckcest.cn/tcm/search/classifybrowse?type=pre#), and the latter were derived from a famous book «Great Dictionary of Chinese Medicine» (https://pan.baidu.com/s/1c14N27Y). Examples of formula data and herb data are listed in Tables 2 and 3.

### 3.1. Setup

In compatibility rule mining step, our method returned top-*N* herb pairs according to their associated Cor value, which was used to decide the parameter *x*_*i*_ during the process of topic modeling. The parameters *a* and *b* in (3) were both set to 0.5 through repeated experiments.

In topic modeling-based method, we set the number of topics *K* to be the number of function labels, which were set to 20. The number of unique herbs extracted from 3055 formulae was 972. Moreover, we set the hyperparameters *α* = 50/*K* and *η* = 0.1 and the iteration number *l* = 500.

In multilabel classifier-based method, we combined the TFIDF feature space and attribute features to represent a formula. The dimension for TFIDF feature space $\vec{\text{p}}$ was set to 972, the number of unique herbs. The dimension for attribute features $\vec{\text{V}}$ was 134. Then, the resulting feature vector of each formula was 1106. We adopted several classifiers (SVM, Adaboost, and Bayes Network) using 4-fold cross validation on 3055 formulae.

We designed five experiments to conduct our prediction task:

- Topic modeling based on Labeled LDA
- Topic modeling based on LPH
- TFIDF feature space
- Attribute feature space
- TFIDF + attribute feature space.

For experiments (a) and (b), we calculated the probability *p*(*k*∣**H** ^(*p*)^) for the new prescription *p*, where *k* ∈ [1 ⋯ *K*]. The label *k* was returned when it satisfied the following condition:

(15) $$\begin{array}{c} p\left(\;k\;|\;H^{\left(p\right)}\;\right)>T, \end{array}$$

where *T* was the threshold. For experiments (c)~(e), these feature vectors were generated and used as inputs to classifiers. We tuned the SVMs' shared cost parameter* C* (=10). The “TFIDF + attributes” features were denoted as $\vec{\text{p}}\cup \vec{\text{V}}$. The prediction was considered as a 20-class, multilabel classification problem. Each test was performed 10 times to obtain the average performance. We scored each method based on Precision, Recall, and Micro-*F*1 as our evaluation measures. These measures were defined as follows:

(16) Precision=The  total  number  of  correct  labels  predicted  by  a  methodThe  total  number  of  labels  predicted  by  a  method,

(17) Recall=The  total  number  of  correct  labels  predicted  by  a  methodThe  total  number  of  real  labels,

(18) Micro-F1=2×Precision×RecallPrecision+Recall.

### 3.2. Experimental Result

#### 3.2.1. Compatibility Rule Mining

We use Precision@*N* metric to evaluate the effectiveness of our method and then determine the number of returned herb pairs. Precision@*N* is the ratio of correct pairs to the* N* returned pairs. The returned pairs are assumed to be correct when they have compatibility rule according to expert's instructions. The experimental results are shown in Table 4.

**Figure 3:**
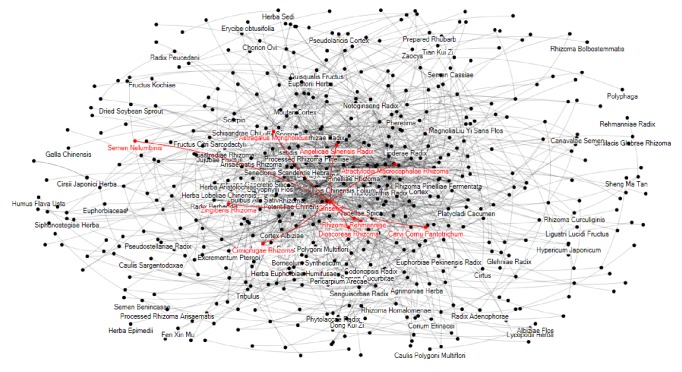
Detected 1500 pairs of herbs.

Based on the above results, when the number of returned pairs is more than 1500, the correct sample does not show an obvious increase. Thus, top 1500 herb pairs are returned in our experiment. The mining results are visualized in Figure 3. Each vertex in the graph represents a herb. An edge is drawn between a pair of herbs if they have compatibility rule. As shown in Figure 3, one herb can have compatibility rule with several other herbs. For instance,* Ginseng Radix* can be combined with* Atractylodis Macrocephalae Rhizoma*,* Zingiberis Rhizoma*,* Dioscoreae Rhizoma*,* Angelicae Sinensis Radix, *or* Cervi Cornu Pantotrichum *to promote different treatment effects. It is clear that utilizing powerful computers and efficient algorithms can mine latent compatibility rules, which would be useful for TCM practitioners for further study.

#### 3.2.2. Topic Discovery

Tables 5 and 6 show the 4 topics detected by LPH model, Table 7 shows the 2 topics detected by Labeled LDA model. Each topic contains top 20 herbs. As shown in Tables 5 and 6, we notice that most of the top 20 herbs have related functions corresponding to the topic, but several detected herbs do not have corresponding function, such as* Plantaginis Semen* in “cleaning heat” topic and* Glycyrrhizae Radix* in “relieving uneasiness of mind” topic. Although* Plantaginis Semen* has low posterior probability and does not have direct correspondence to the topic, the herb is an important component in some prescriptions having the corresponding function.* Glycyrrhizae Radix *can be detected in most of topics, since it is frequently used in many formulae to regulate actions of all other herbs. It has to be noted that* Glycyrrhizae Radix* is removed from the combinational rule mining results (see Section 2.1.1), not the topic modeling results; thus it can be assigned to a topic (function) as a single herb in the results of topic discovery.

In other topics, we can find similar results as well. Most of the herbs (marked by the rectangle) that do not have intensive correlation with the topic have low probability. A pair of herbs tend to indicate more intensive correlation with the corresponding topics than a single herb, such as* Ginseng Radix* and* Atractylodis Macrocephalae Rhizoma* from “relieving uneasiness of mind” topic and* Atractylodis Macrocephalae Rhizoma *and* Angelicae Sinensis Radix* from “dispelling internal cold” topic. Therapeutic effects can be promoted by the coordination of two herbs. In addition, many individual herbs are inactive in the corresponding topic but become active in combination with other herbs, such as* Paeoniae Radix Alba* and* Szechwan Lovage Rhizome *from “dispelling internal cold” topic. However, Labeled LDA cannot discover combinations of effective interacting herbs (see Table 7).

#### 3.2.3. Function Prediction

In employing the LPH model to solve the multilabel classification problem, we should determine the threshold *T* in (15). However, there is no theoretical basis to automatically choose an optimal threshold. In this study, we provide the experimental results using different thresholds (see Table 8).

Table 9 shows the classification performance. Comparing the above two methods, multilabel classifiers perform slightly better than topic model-based methods. As shown in Table 8, the value of threshold has a strong influence on the classification results. We can take *T* = 1*e* − 8 as an optimal value to achieve optimal prediction power. LPH substantially outperforms Labeled LDA on Micro-*F*1 with the optimal *T*. The results demonstrate that incorporating compatibility rule into topic model can promote prediction accuracy. The recall on both two models are not satisfactory, as the posterior probability can highlight the most probable function labels but neglect others.

From Table 9, we notice that when using TFIDF features only, the performance is not good. The predictive ability based on herbal attributes is better than TFIDF features. This indicates that “channel tropism,” “nature & flavor,” and “efficiency” are valuable information for function prediction, which is consistent with TCM theory. The combination of the features outperforms individual feature space. SVM produces the highest Micro-*F*1 on the “TFIDF + attributes” feature space compared with other classifiers.

### 3.3. Discussion

From the compatibility rule mining results, we can see that our method can effectively discover herb pairs with combinational rules. The method is not meant to perfectly model TCM reality, but to function as a tool for TCM practitioners. Also, it can indicate herbs that are likely to be used together for special therapeutic effects and allow researchers to make attempts at further study.

From the topic discovery results, we can see that it is feasible to employ the supervised topic model to predict the function of a new prescription. The idea of incorporating compatibility rules into the process of topic modeling promotes the accuracy of our task. The results are more satisfactory than Labeled LDA because the efficiency of a pair of herbs is more explicit than a single herb, which contributes to the function prediction on a new prescription.

The two proposed kinds of methods can provide valuable information for new prescription discovery before clinical test procedures [16], but each has its advantages. The method based on multilabel classifiers contains complicated and trivial steps in feature extraction, such as dosage standardization and attributes quantification, while the LPH topic model cannot choose the optimal threshold automatically. Although we may improve the function prediction performance by using SVM classifier and LPH model, the results are not very satisfactory. It is possible to combine these two methods to promote prediction accuracy in our future work.

## 4. Conclusions

This paper has presented two methods for prescription function prediction. In the first method, we employ a novel supervised topic model named LPH to calculate the prescription's mostly likely function labels. In the second method, we extract feature space based on TFIDF weighting and herbal attributes and use these features to build multilabel classifiers. Results on real world datasets show the effectiveness of our methods. The results can provide valuable information for new prescription discovery.

When doctors write a prescription for the patient, they should obey the principal named “Jun,” “Chen,” “Zuo,” “Shi”, which plays a significant role in determining a prescription's function. In the future, we plan to analyze the components of a prescription based on its herbal attributes and dosage information. In other words, the herbs in a prescription may possibly be clustered into four classes by data mining algorithms. The results may further improve the accuracy of our prediction task.

## Figures and Tables

**Table 1:** Dosage standardization for “Ma Huang Tang” (g).

Ma Huang Tang	*d*_*i*_	*d*_min_	*d*_max_	*d*_*i*_^*∗*^
*Ephedrae Herba*	9	2	9	0.82
*Cinnamomi Ramulus *	6	3	9	0.50
*Armeniacae Semen Amarum *	6	4.5	9	0.44
*Glycyrrhizae Radix*	3	1.5	9	0.29

**Table 2:** An example of a formula.

Formula	Ma Huang Tang
Herbs	*Ephedrae Herba *(9 g), *Cinnamomi Ramulus* (9 g), *Armeniacae Semen Amarum* (6 g), *Glycyrrhizae Radix* (3 g)
Function	Relieving exterior syndrome

**Table 3:** The detailed information about *“Ephedrae Herba.”*

Herb	*Ephedrae Herba*
Efficiency	Inducing perspiration, relieving superficies by cooling, opening the inhibited lung-energy, relieving asthma, clearing dam, subsidence of a swelling
Nature & flavor	Spicy, slightly bitter, warm
Channel tropism	Lungs, bladder
Usual dosage	2 g ~ 9 g

**Table 4:** Experimental results of compatibility rule mining.

Number of returned herb pairs	Precision@*N*	Number of returned herb pairs	Precision@*N*
100	100/100	1000	913/1000
200	200/200	1100	974/1100
300	294/300	1200	1026/1200
400	383/400	1300	1078/1300
500	472/500	1400	1135/1400
600	550/600	1500	1166/1500
700	630/700	1600	1171/1600
800	711/800	1700	1173/1700
900	809/900	1800	1174/1800

**Table 5:** Topics discovered by LPH model.

Cleaning heat	Probability	Relieving uneasiness of mind	Probability
*Szechwan Lovage Rhizome*, *Angelicae Sinensis Radix*	0.05953	*Polygalae Radix *	0.04842
*Unprocessed Rehmanniae Radix *	0.05431	*Ginseng Radix, Atractylodis Macrocephalae Rhizoma *	0.03805
*Atractylodis Macrocephalae Rhizoma, Paeoniae Radix Alba*	0.03238	*Rhei Radix *	0.03574
*Scutellariae Radix *	0.02507	*Jujubae Fructus *	0.03259
*Paeoniae Radix Alba*	0.02403	Glycyrrhizae Radix	0.02017
*Phellodendri Chinensis Cortex, Anemarrhenae Rhizoma *	0.02403	*Angelicae Sinensis Radix *	0.01960
*Glycyrrhizae Radix*	0.02298	*Poria, Szechwan Lovage Rhizome*	0.01615
*Poria *	0.02194	*Fossil Fragments,Ostreae Concha*	0.01615
*Rehmanniae Radix *	0.01881	*Zingiberis Rhizoma*	0.01384
*Coptidis Rhizoma*	0.01776	*Coptidis Rhizoma*	0.01384
*Dichroae Radix *	0.01672	*Acori Tatarinowii Rhizoma*	0.01038
*Ophiopogonis Radix *	0.01567	*Fresh Rehmanniae Radix*	0.01038
*Forsythiae Fructus *	0.01463	*Kansui Radix*	0.01038
*Cimicifugae Rhizoma, Clerodendron Cyrtophyllum Turcz *	0.01254	*Dried Rehmanniae Radix*	0.01038
*Ginseng Radix*	0.01254	*Aconiti Lateralis Radix Praeparata, Pinelliae Rhizoma*	0.01038
Plantaginis Semen	0.01254	*Schisandrae Chinensis Fructus*	0.01038
*Saposhnikoviae Radix, Notopterygii Rhizoma *	0.01254	Realgar	0.00923
*Ostreae Concha *	0.01150	*Salviae Miltiorrhizae Radix*	0.00923
*Mume Fructus *	0.01150	Saposhnikoviae Radix	0.00923
*Cinnamomi Ramulus*, *Paeoniae Radix Alba *	0.00856	*Scrophulariae Radix*	0.00923

**Table 6:** Topics discovered by LPH model.

Replenishing and restoring	Probability	Dispelling internal cold	Probability
*Atractylodis Macrocephalae Rhizoma Ginseng Radix*	0.05533	*Zingiberis Rhizoma Recens*	0.04842

*Poria, Szechwan Lovage Rhizome*	0.05297	Glycyrrhizae Radix	0.03805

*Astragali Radix *	0.03708	*Codonopsis Radix *	0.03574

*Angelicae Sinensis Radix, Dioscoreae Rhizoma *	0.03120	*Pinelliae Rhizoma, Poria*	0.03459

Glycyrrhizae Radix	0.02767	*Atractylodis Macrocephalae Rhizoma, Angelicae Sinensis Radix *	0.02421

*Codonopsis Radix *	0.02649	*Astragali Radix *	0.01960

*Rehmanniae Radix Praeparata, Angelicae Sinensis Radix *	0.02531	*Cinnamomi Ramulus *	0.01615

*Paeoniae Radix Alba *	0.02096	*Paeoniae Radix Alba, Szechwan Lovage Rhizome*	0.01499

*Pinelliae Rhizoma *	0.01325	*Fossil Fragments, Ostreae Concha*	0.01384

*Dried Rehmanniae Radix *	0.01325	*Leonuri Herba *	0.01384

*Asini Corii Colla, Angelicae Sinensis Radix *	0.00943	*Asini Corii Colla, Angelicae Sinensis Radix *	0.01384

*Schisandrae Chinensis Fructus, Atractylodis Macrocephalae Rhizoma*	0.00943	*Ginseng Radix*	0.01269

*Asari Radix, Zingiberis Rhizoma *	0.00943	Atractylodis Rhizoma	0.01154

*Cornu Cervi Pantotrichum *	0.00943	Radix Asparagi	0.01038

*Salviae Miltiorrhizae Radix, Schisandrae Chinensis Fructus *	0.00943	*Saposhnikoviae Radix, Angelicae Pubescentis Radix *	0.01038

*Zingiberis Rhizoma Recens *	0.00825	*Zingiberis Rhizoma *	0.01038

*Polygalae Radix *	0.00825	Platycodonis Radix	0.00923

*Poria*	0.00825	*Salviae Miltiorrhizae Radix *	0.00923

Gastrodiae Rhizoma	0.00825	*Ephedrae Herba *	0.00923

*Sophorae Flavescentis Radix *	0.00707	Aconiti Lateralis Radix Praeparata	0.00820

**Table 7:** Topics discovered by Labeled LDA model.

Cleaning heat	Probability	Relieving uneasiness of mind	Probability
*Unprocessed Rehmanniae Radix *	0.03172	*Polygalae Radix *	0.04112
*Glycyrrhizae Radix*	0.02984	Glycyrrhizae Radix	0.03945
*Szechwan Lovage Rhizome*	0.02773	*Ginseng Radix*	0.03712
*Ophiopogonis Radix*	0.02678	*Salviae Miltiorrhizae Radix *	0.03226
*Scutellariae Radix *	0.02421	*Rhei Radix *	0.03226
*Moutan Cortex *	0.01933	*Jujubae Fructus *	0.02110
*Anemarrhenae Rhizoma *	0.01933	*Angelicae Sinensis Radix *	0.02110
*Atractylodis Macrocephalae Rhizoma *	0.01847	*Fresh Rehmanniae Radix *	0.02110
*Rehmanniae Radix*	0.01847	*Poria *	0.01958
*Paeoniae Radix Alba *	0.01847	*Scrophulariae Radix *	0.01646
*Ginseng Radix*	0.01811	*Coptidis Rhizoma*	0.01617
*Coptidis Rhizoma*	0.01652	*Zingiberis Rhizoma *	0.01617
*Forsythiae Fructus *	0.01584	*Kansui Radix *	0.01617
Cinnamomi Ramulus	0.01437	*Fossil Fragments *	0.01025
*Phellodendri Chinensis Cortex *	0.01437	*Acori Tatarinowii Rhizoma *	0.00943
*Saposhnikoviae Radix *	0.01394	*Aconiti Lateralis Radix Praeparata *	0.00943
*Mume Fructus *	0.01394	*Pinelliae Rhizoma *	0.00943
*Poria*	0.01386	*Dried Rehmanniae Radix *	0.00872
*Chinese Herbaceous Peony *	0.00945	*Lycii Fructus*	0.00845
*Ostreae Concha *	0.00835	Realgar	0.00845

**Table 8:** Average performance of topic model-based method.

Threshold *T*	Labeled LDA			LPH		
	Precision	Recall	Micro-*F*1	Precision	Recall	Micro-*F*1
1*e* − 5	0.6102	0.1187	0.1987	0.8124	0.1025	0.1820
1*e* − 6	0.7317	0.2658	0.3899	0.6075	0.2031	0.3044
1*e* − 7	0.6567	0.3278	0.4373	0.6874	0.3295	0.4455
1*e* − 8	0.5927	0.4076	0.4830	**0.7220**	**0.4187**	0.5300
1*e* − 9	0.5365	0.4127	0.4665	0.6267	0.4203	0.5031

**Table 9:** Average performance of multilabel classifiers.

Classifier	Feature space	Precision	Recall	Micro-*F*1
SVM	TFIDF	0.6202	0.3945	0.4822
	Attributes	0.6510	0.4102	0.5033
	TFIDF + attributes	**0.7359**	0.4823	**0.5827**

Adaboost	TFIDF	0.5729	0.3102	0.4025
	Attributes	0.6856	0.3358	0.4508
	TFIDF + attributes	0.6894	0.3475	0.4621

Bayes Network	TFIDF	0.5126	0.4325	0.4691
	Attributes	0.6179	0.4218	0.5013
	TFIDF + attributes	0.6397	0.5124	0.5690
